# Increased Risk of Vitamin D Deficiency Among HIV-Infected Individuals: A Systematic Review and Meta-Analysis

**DOI:** 10.3389/fnut.2021.722032

**Published:** 2021-08-18

**Authors:** Yingying Wang, Xiaojie Huang, Yaxin Wu, Aixin Li, Yakun Tian, Meixin Ren, Zhen Li, Tong Zhang, Hao Wu, Wen Wang

**Affiliations:** Center for Infectious Diseases, Beijing Youan Hospital, Capital Medical University, Beijing, China

**Keywords:** HIV, vitamin D deficiency, ART, prevalence, human inmunodeficiency virus

## Abstract

**Background:** Human immunodeficiency virus (HIV) infection is a heavy burden worldwide. Observational studies have reported a high prevalence of vitamin D deficiency (VDD) among people living with HIV (PLWH). However, its deficiency is also a global health problem. Therefore, we conducted a meta-analysis and systemic review to compare differences between HIV-infected subjects and non-HIV-infected subjects.

**Methods:** We searched PubMed, Web of Science, Embase, and Cochrane library. We extracted data, including demographic information, study type, vitamin D-related values, and HIV-related values, ultimately including 15 studies after removing duplicates and screening titles, abstracts, and full texts and finally performing a meta-analysis in terms of vitamin D level and vitamin D deficiency prevalence.

**Results:** Regarding VDD prevalence, the HIV vs. the non-HIV group had an odds ratio of 1.502 (95% CI, 1.023–2.205; *P* = 0.038). In the subgroup analysis, the odds ratios were 1.647 (95% CI, 1.020–2.659; *P* = 0.041; *I*^2^ = 94.568) from 7 studies (age over 40), 2.120 (95% CI, 1.122–4.008; *P* = 0.021; *I*^2^ = 0.000) from 2 studies (BMI less than or equal to 25), 1.805 (95% CI, 1.373–2.372; *P* = 0.042; *I*^2^ = 74.576) from 7 studies (latitude <40), 2.120 (95% CI, 1.122–4.088; *P* = 0.021; *I*^2^ = 0.000) from 2 studies (only included male participants), and 2.296 (95% CI, 1.287–4.097; *P* = 0.005; *I*^2^ = 19.927) from 3 studies (only included ART-experienced participants). Thirteen studies were deemed to have moderate quality, while two had high quality.

**Conclusions:** HIV infected subjects are prone to have VDD compared with general population. ART, older age, lower BMI, lower latitude and male sex may present risk factors for VDD in PLWH.

**Systematic Review Registration:**https://www.crd.york.ac.uk/PROSPERO/display_record.php?RecordID=228096.

## Introduction

Human immunodeficiency virus (HIV) infection is a global concern. Thirty-eight million people are living with HIV (PLWH); however, only 26 million receive treatment. The goal of 90−90−90, launched by the Joint United Nations Programme on HIV/AIDS (UNAIDS), was still underachieved in 2019. Fortunately, the deaths related to HIV have decreased by 39% since 2010^1^. People must receive antiretroviral therapy (ART) since there is still no cure (1). Recent guidelines for first-line therapy are different among the USA, Europe and WHO. The Nucleoside reverse transcriptase inhibitor (NRTI)-based regimen plus DTG is recommended as the preferred first-line regimen by the WHO (2). In contrast, the USA suggests an inhibitor-based strategy (3). Tenofovir Disoproxil Fumarate (TDF) or Tenofovir alafenamide (TAF), plus one of which includes rilpivirine, boosted darunavir, and an integrase inhibitor, are allowed in Europe (4). Improvements are apparent in terms of viral suppression, quality of life, life span, and so on (3). Simultaneously, chronic diseases involving bone, kidney, and heart and metabolic disorders such as vitamin D deficiency, have appeared (5).

Vitamin D, a steroid hormone that balances homeostasis, involves bone metabolism and calcium absorption, affecting many processes in the human body. Vitamin D metabolites have been recognized to support innate antiviral mechanisms, including antimicrobial peptides and autophagy. Immune cells, which include monocytes and macrophages, are equipped to synthesize and respond to active vitamin D (6). These cells could have an antibacterial effect on Vitamin D receptor-expressing cells, such as T cells and B cells, by releasing 1,25(OH)_2_D in response to the induction by pathogens, such as M. tuberculosis (6). In addition, vitamin D receptors exist in broad tissues, including the heart muscle, kidney, cancer cells, lungs, and so on (7). Observational and ecological studies have demonstrated correlations between low serum vitamin D concentration and increased risk of cardiovascular disease, cancer, diabetes, and death (8).

Vitamin D deficiency may cause immune dysfunction by altering the expression of autophagy and inflammatory markers in HIV-infected subjects. Additionally, its deficiency is also linked with an increased risk of AIDS-related comorbidities and mortalities (9). Recently, vitamin D has attracted attention in many fields. Its supplementation strengthens muscle, lowers the fracture risk in elderly patients (10), reduces chronic obstructive pulmonary disease exacerbations, and prevents respiratory tract infections in children (11, 12). Furthermore, it reduces the coinfection and progression of HIV/TB (13). However, vitamin D deficiency is a common concern. Approximately 7% of the population presents with <30 ng/ml of serum vitamin D concentrations worldwide (14). Approximately 5 and 14% of individuals live with severe vitamin D deficiency in the USA and Europe, respectively. At the same time, the Middle East or Gulf states reported an even higher percentage (15).

Recent studies reported higher vitamin D deficiency prevalence in PLWH (16–18). Many factors, such as HIV infection, ART, detectable HIV RNA, and HIV-related comorbidities, could be the reason for hypovitaminosis D (19). For example, efavirenz (EFV) enhances the transformation of active vitamin D into inactive forms by inducing the cytochrome P450 enzyme (20). Simultaneously, the inconsistency of whether protease inhibitor (PI) or TDF leads to a lower vitamin D levels is still unresolved. A cross-sectional study showed that PI-boosted monotherapy was related to a lower risk of vitamin D deficiency by inhibiting vitamin D activation (21), but Cervero et al. reported the opposite result (22).

Many studies have estimated the prevalence of vitamin D deficiency. However, a consensus on whether HIV-infected subjects exhibit serum vitamin D concentrations or higher vitamin D deficiency prevalence than non-HIV-infected populations has not been reached. Therefore, we aimed to investigate the difference in both outcomes between HIV and control groups.

## Materials and Methods

### Study Design

We performed a systematic review and meta-analysis based on PRISMA guidelines, and it was registered in PROSPERO (CRD42021228096).

### Data Sources and Searches

PubMed, Embase, Web of Science, and the Cochrane Library were researched for relevant articles.

We used the Medical Subject Heading terms for PubMed and comparable terms for other databases. Search terms included vitamin D (“vitamin D,” “vitamin D deficiency,” “25-hydroxyvitamin D,” “calcifediol,” “ergocalciferols,” and “cholecalciferol”) and HIV (“HIV” and “HIV Infections”). The detailed search strategy is described in Supplementary Table 1. Studies that fulfilled both the inclusion and exclusion criteria published before November 29, 2020 were included.

### Study Selection

The inclusion criteria were as follows: (1) human study related to HIV, (2) participants over 18 y of age diagnosed with HIV, (3) observational study with a comparable non-HIV healthy or general control group, and (4) accessible data examining the frequency of VDD or the levels of VD in each group.

Exclusion criteria included the following: (1) experimental studies, (2) intervention studies, (3) no control group for comparison or unclear information for the control group, (4) conference proceedings and abstracts, short or brief communications, (5) case reports or case series, (6) non-English language studies, (7) participants included pregnant women, and (8) human studies related to tuberculosis.

All the studies searched from four database were sent to citation manager (Endnote X9). After removing duplicates by using the citation manager, researchers (Y.Y.W. and Y.X.W.) read through the titles and abstracts of the studies independently. Full texts were obtained, and further screening was performed when the studies were recognized as eligible or uncertain with respect to their eligibility. Disagreements were resolved by consensus.

### Data Extraction

Two authors independently extracted data using predefined and standardized data extraction forms, combining the data if studies included several subgroups. Disagreements were resolved by discussion. The extracted information included: year of publication, name of the first author, sample size, study design, sampling date or season, male proportion, country, method of vitamin D measurement, mean serum vitamin D level, vitamin D deficiency prevalence, and HIV-related information.

### Risk of Bias

Researchers assessed the quality of the included studies, case-control studies and cohort studies using the Newcastle–Ottawa Scale. Cross-sectional studies were evaluated using the scale launched by the Agency for Healthcare Research and Quality (23). Selection and comparability were both considered in case-control and cohort studies. At the same time, exposure was evaluated in case-control studies, and the outcome was evaluated in cohort studies. The highest score is 9. A high risk of bias was deemed when the score was <5, 5–7 was deemed to be moderate, and <7 was considered a low risk of bias. Eleven items were judged in the cross-sectional study. Publication bias was assessed according to a visualized funnel plot. Low publication bias appeared when the plot was symmetrical.

### Outcomes

The primary outcomes for this review were differences in serum 25 (OH) D levels and vitamin D deficiency prevalence between participants with and without HIV infection.

### Statistical Analysis

Search results were sent to citation manager software. We performed statistical analysis on pooled mean serum 25 (OH) D concentration and vitamin D prevalence using CMA software. Heterogeneity was expected to be high due to numerous factors influencing vitamin D syntheses, such as season, ethnicity, and latitude. Consequently, we used the random-effects model to conduct data synthesis. Heterogeneity was assessed using the *I*^2^ test, we considered values below 25% to be low, 25–50% to be moderate and over 50% to be high, and it was considered substantial when <75% (24). A significant difference was considered with a *p*-value <0.05.

#### Data Synthesis

We transformed data using the formula SD = SE x √ N when presented in the form of the mean ± SE (25). N represents the sample size or the number of participants. When data was provided as the median ± IQR, they were converted into estimated mean ± SD using an established method (26). Data presented only with medians were excluded from the final meta-analysis. Data needed to be pooled based on the Cochrane Handbook for Systematic Reviews of Interventions if provided as the mean ± SD from two or more subgroups (27). Serum vitamin D levels were converted into ng/ml by dividing by 2.5 when they were presented as nmol/L (14).

#### Subgroup Analysis and Meta-Regression

We analyzed predefined subgroups (28) by age (>40 or ≤40), latitude (>40 or ≤40), ART (HIV participants with 100% ART or without ART), HIV RNA (detectable vs. undetectable), BMI (>25 vs. ≤ 25), and sex (only male vs. only female) to determine the factors affecting heterogeneity.

We also adopted the variables (the proportion of ART, male proportion, Undetectable HIV RNA proportion, BMI, latitude, publication year, and CD4 count) to perform a meta-regression to find out the sources of heterogeneity.

## Results

### Literature Search Results

**Figure 1:**
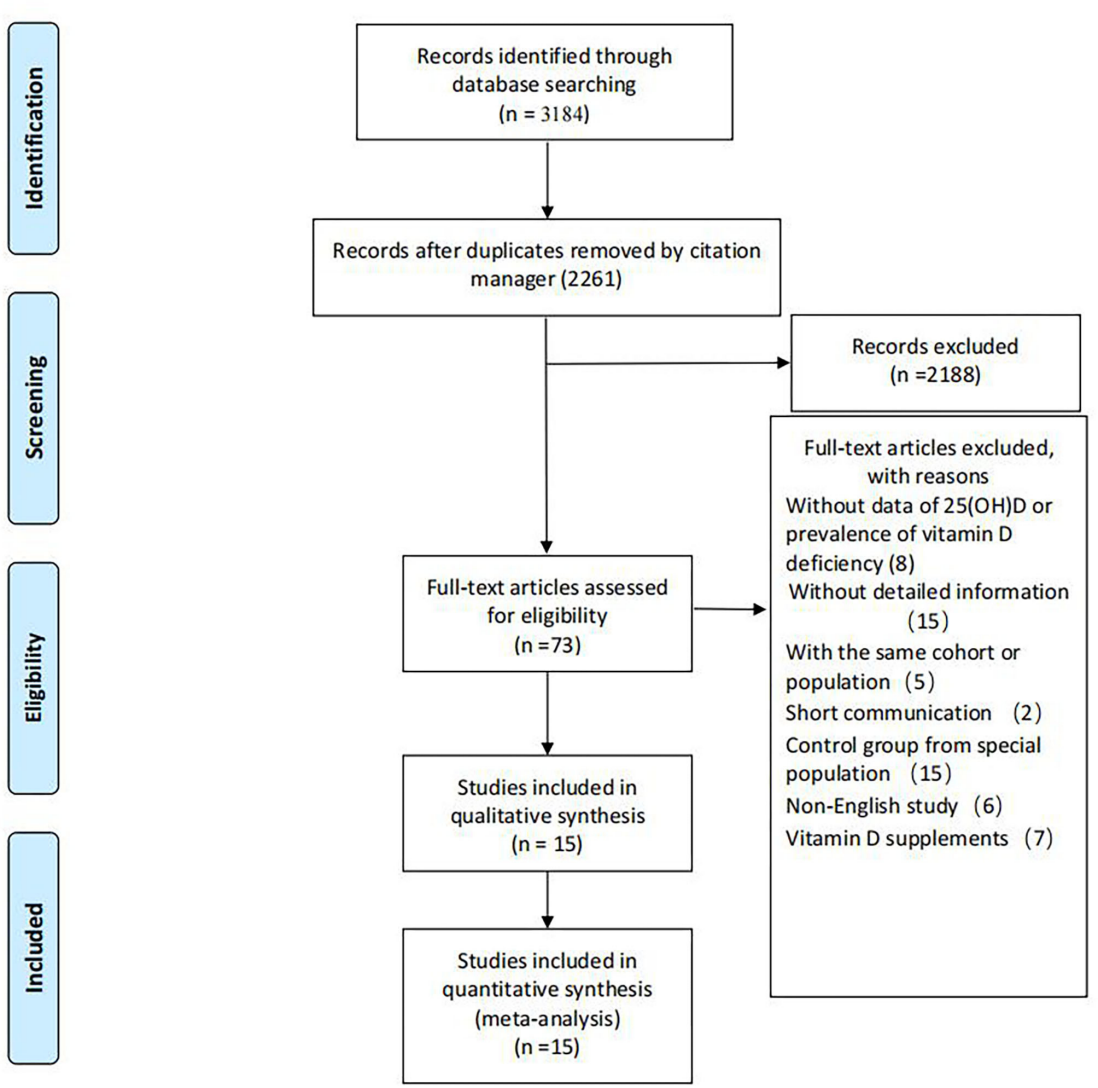
Flowchart of the studies included in the meta-analysis.

A total of 2,261 records were screened for titles and abstracts after the initial removal of 923 duplicates by the citation manager. Seventy-three articles were left for full-text screening. Fifteen studies were included in our review. Details for the progress of searching are available in Figure 1.

Six studies were performed in the United States (18, 29–33), 2 in Italy (34, 35), and 1 each from China (36), Chile (37), Iran (28), Australia (38), Poland (39), Brazil (40), and India (41). Seven studies recruited participants based on ART status, 4 of which included ART-experienced HIV subjects (29, 33, 35, 39), while the rest included ART-naïve HIV-infected subjects (31, 36, 37). Three studies were performed in the male population (33, 37, 41), whereas 1 study included only females (18). Twenty-five (OH) D was detected using enzyme-linked immunosorbent assay (ELISA) (28, 29), high-performance liquid chromatography (34, 35), Ultra-HPLC-tandem MS (30), immunoaffinity-liquid chromatography-tandem mass spectrometry (33), Electrochemiluminescence immunoassay (36), liquid chromatography/mass spectrometry (18, 32, 37), chemiluminescent assay (31, 38), luminescent method (39), chemiluminescent microparticle immunoassay (40), and radioimmunoassay (41). The primary characteristics of the included studies are shown in Tables 1, 2. The risk of bias from the included articles is presented in Tables 3, 4.

**Table 1:** The characteristics of included studies for assessing vitamin D level.

**References**	**Country**	**Study design**	**Latitude**	**HIV age (year)**	**Control age (year)**	**Male percentage**	**BMI**	**Sample size**	**HIV number**	**Control number**	**VD level in HIV**	**VD level in control**	***P*-value**
Ross et al. (29)	USA	Cross-sectional study	41°30′N	49	38	80.9	26.19	183	149	34	25.84 (0.432)	20.84 (0.416)	<0.001
Atteritano et al. (34)	Italy	Case-control study	38°13′N	45.36	44.85	16	24.9	200	100	100	20.29 (4.05)	35.77 (6.5)	<0.001
Thuppal et al. (30)	USA	Cross-sectional study	multicenter	37.7	34.3	50	28.3	15792	85	15707	21.793 (13.74)	25.461 (28.36)	0.233
Zhang et al. (36)	China	Cohort study	39°55′N	37.3	37.19	87.5	22.88	80	40	40	15.93 (6.49)	19.12 (9)	0.069
Zhang et al. (33)	USA	Cohort study	multicenter	NA	NA	100	NA	658	466	192	18.5 (8.889)	18 (6.593)	0.482
Adeyemi et al. (18)	USA	Cross-sectional study	multicenter	44	41	0	28.57	1778	1268	510	16 (11.111)	14 (8.148)	<0.001
Ceballos et al. (37)	Chile	Cross-sectional study	33°26′S	31.2	33.3	100	24.44	91	70	21	17.7 (6.64)	20.8 (7)	0.064
Currò et al. (35)	Italy	Case-control study	38°13′N	43.2	41.5	59.8	NA	97	57	40	23.96 (10.871)	34.96 (12.396)	<0.001
Hileman et al. (31)	USA	Cohort study	41°30′N	40	37	69.3	26.65	88	47	41	13.245 (8.258)	15.1 (6.81)	0.255
Janbakhsh et al. (28)	Iran	Case-control study	34°19′N	40.11	45.59	87.2	NA	196	98	98	29.56 (27.27)	30.63 (18.66)	0.749
Klassen et al. (38)	Australia	Cross-sectional study	37°49′S	41	50	56	NA	4650	997	3653	24.8 (11.852)	27.6 (9.185)	<0.001
Mikula et al. (39)	Poland	Cross-sectional study	52°15′N	44.9	56.6	73.9	NA	188	148	40	29.68 (14.36)	30 (11.04)	0.896
Flauzino et al. (40)	Brazil	Cross-sectional study	23°30′S	40	40	53.5	25.17	441	314	127	29.94 (11.695)	29.07 (9.128)	0.453
Paul et al. (41)	India	Cross-sectional study	12°55′N	38.45	38.6	100	23.13	105	70	35	19.7 (8.804)	22.2 (5.6)	0.126

*NA, not available; N: north; S: south; P-value: for evaluating the difference in VD levels between HIV and control groups*.

**Table 2:** Characteristic of included studies for assessing the Vitamin D Deficiency prevalence.

**References**	**Country**	**Study design**	**Latitude**	**HIV age (year)**	**Control age (year)**	**Male percentage**	**BMI**	**No. of HIV group**	**No. of control group**	**No. of VDD in HIV group**	**No. of VDD in control group**	***P*-value**
Ross et al. (29)	USA	Cross-sectional study	41°30′N	49	38	80.9	26.19	149	34	69	10	0.076
Adeyemi et al. (18)	USA	Cross-sectional study	multicenter	44	41	0	28.57	1,268	510	758	366	<0.001
Ceballos et al. (37)	Chile	Cross-sectional study	33°26′S	31.2	33.3	100	24.44	70	21	46	10	0.139
Currò et al. (35)	Italy	Case-control study	38°13′N	43.2	41.5	59.8	NA	57	40	25	6	0.004
Hidron et al. (32)	USA	Cross-sectional study	33°46′N	50	63	88.5	26	933	5,355	495	2,062	<0.001
Hileman et al. (31)	USA	Cohort study	41°30′N	40	37	69.3	26.65	47	41	33	32	0.405
Janbakhsh et al. (28)	Iran	Case-control study	34°19′N	40.11	45.59	87.2	NA	98	98	44	35	0.191
Klassen et al. (38)	Australia	Cross-sectional study	37°49′S	41	50	56	NA	997	3,653	398	805	<0.001
Mikula et al. (39)	Poland	Cross-sectional study	52°15′N	44.9	56.6	73.9	NA	148	40	36	7	0.364
Flauzino et al. (40)	Brazil	Cross-sectional study	23°30′S	40	40	53.5	25.17	314	127	178	75	0.649
Paul et al. (41)	India	Cross-sectional study	12°55′N	38.45	38.6	100	23.13	70	35	39	13	0.075

*NA, not available; N, north; S, south; P-value: for evaluating the difference in VD deficiency prevalence between HIV and control groups; No: number*.

**Table 3:** Quality assessment of included studies.

**References**	**Study type**	**Selection**				**Comparability**	**Exposure/Outcome**			**Final score**
		**Item 1**	**Item 2**	**Item 3**	**Item 4**	**Item 5**	**Item 6**	**Item 7**	**Item 8**	
Curro et al. (35)	Case-control study	1	0	1	1	1	0	1	0	5
Atteritano et al. (34)	Case-control study	1	1	1	1	2	0	1	0	7
Janbakhsh et al. (28)	Case-control study	1	0	1	1	1	0	1	0	5
Zhang et al. (33)	Cohort study	0	1	1	1	1	1	1	0	6
Zhang et al. (36)	Cohort study	1	1	1	1	1	1	1	1	8
Hileman et al. (31)	Cohort study	1	0	0	1	1	1	1	1	6

*For case-control study; Item 1 Is the case definition adequate? Item 2 Representativeness of the cases; Item 3 Selection of Controls; Item 4 Definition of Controls; Item 5 Comparability of cases and controls on the basis of the design or analysis; Item 6 Ascertainment of exposure; Item 7 Same method of ascertainment for cases and controls; Item 8 Non-Response rate*.

*For cohort study; Item 1 Representativeness of the exposed cohort; Item 2 Selection of the non-exposed cohort; Item 3 Ascertainment of exposure; Item 4 Demonstration that outcome of interest was not present at start of study; Item 5 Comparability of cohorts on the basis of the design or analysis; Item 6 Assessment of outcome; Item 7 Was follow-up long enough for outcomes to occur; Item 8 Adequacy of follow up of cohorts*.

**Table 4:** Quality assessment of included cross-sectional studies.

**References**	**Item 1**	**Item 2**	**Item 3**	**Item 4**	**Item 5**	**Item 6**	**Item 7**	**Item 8**	**Item 9**	**Item 10**	**Quality**
Adeyemi et al. (18)	Yes	Yes	Yes	Yes	No	No	No	Yes	Unclear	No	Moderate
Klassen et al. (38)	Yes	Yes	Yes	Yes	No	Yes	No	No	Unclear	Yes	Moderate
Mikula et al. (39)	Yes	Yes	No	No	No	No	No	No	Unclear	Yes	Moderate
Flauzino et al. (40)	Yes	No	No	Yes	No	Yes	No	Yes	Unclear	No	Moderate
Paul et al. (41)	Yes	Yes	No	Unclear	No	No	No	Yes	Unclear	No	Moderate
Ross et al. (29)	Yes	Yes	Yes	Unclear	No	Yes	No	Yes	Unclear	No	Moderate
Thuppal et al. (30)	Yes	Yes	Yes	Unclear	No	Yes	No	Yes	Unclear	No	Moderate
Ceballos et al. (37)	Yes	Yes	Yes	Unclear	No	No	No	Yes	Unclear	No	Moderate
Hidron et al. (32)	Yes	No	Yes	Yes	No	No	No	Yes	Unclear	No	Moderate

*The 10 evaluation items are as follows:*

*Item 1 Define the source of information (survey, record review).*

*Item 2 List the inclusion and exclusion criteria for exposed and unexposed subjects (cases and controls) or refer to previous publications.*

*Item 3 Indicate the time period used for identifying patients.*

*Item 4 Indicate whether or not the subjects were consecutive, if not population-based.*

*Item 5 Indicate if the evaluators of the subjective components of the study were masked to other aspects of the status of the participants.*

*Item 6 Describe any assessments undertaken for quality assurance purposes (e.g., test/retest of primary outcome measurements).*

*Item 7 Explain the exclusion of any patient from the analysis.*

*Item 8 Describe how confounding variables were assessed and/or controlled.*

*Item 9 If applicable, explain how missing data were handled in the analysis.*

*Item 10 Summarize the patient response rates and completeness of the data collection*.

### Differences in Vitamin D Deficiency Prevalence Between HIV and Control Groups

**Figure 2:**
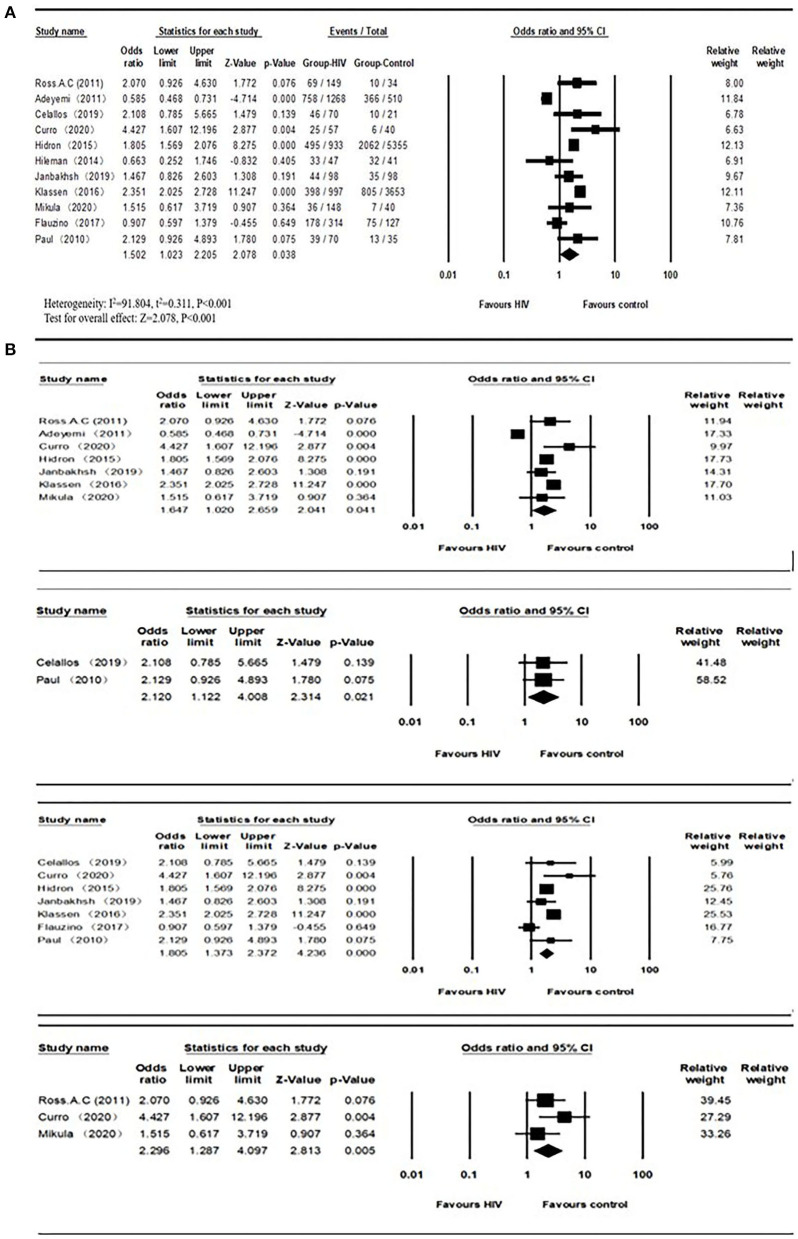
**(A)** Vitamin D deficiency prevalence in HIV and control groups; **(B)** Subgroups of vitamin D deficiency (Age > 40, BMI ≤ 25, Latitude < 40, ART-experienced studies from top to bottom) prevalence between HIV and control groups.

The overall number of VDD and sample size of HIV and the control group from 11 studies were combined and calculated. The overall odds ratio (OR) for HIV vs. the control group was 1.502 (95% CI, 1.023–2.205; *P* = 0.038), with substantial heterogeneity (*I*^2^ = 91.804). In subgroup analyses, the overall ORs were 1.647 (95% CI, 1.020–2.659; *P* = 0.041; *I*^2^ = 94.568) from 7 studies (age over 40), 2.120 (95% CI, 1.122–4.008; *P* = 0.021; *I*^2^ = 0.000) from 2 studies (BMI less than or equal to 25), 1.805 (95% CI, 1.373–2.372; *P* = 0.042; *I*^2^ = 74.576) from 7 studies (latitude <40), 2.120 (95% CI, 1.122–4.088; *P* = 0.021; *I*^2^ = 0.000) from 2 studies (only included male participants), and 2.296 (95% CI, 1.287–4.097; *P* = 0.005; *I*^2^ = 19.927) from 3 studies (only included ART-experienced participants). We did not observe a biased correlation from Begg and Mazumdar rank collection test results. Egger's regression showed that the intercept was 0.75804 (95% CI, −4.59309 to 3.07702; *t* = 0.44714), and the *P*-values were 0.33267 and 0.66534 in one-tailed and two-tailed analyses, respectively. The forest plots are shown in Figure 2. Detailed subgroup results and the funnel plots are shown in Supplementary Table 2 and Supplementary Figure 1.

### Differences in Serum Vitamin D Levels Between HIV and Control Groups

**Figure 3:**
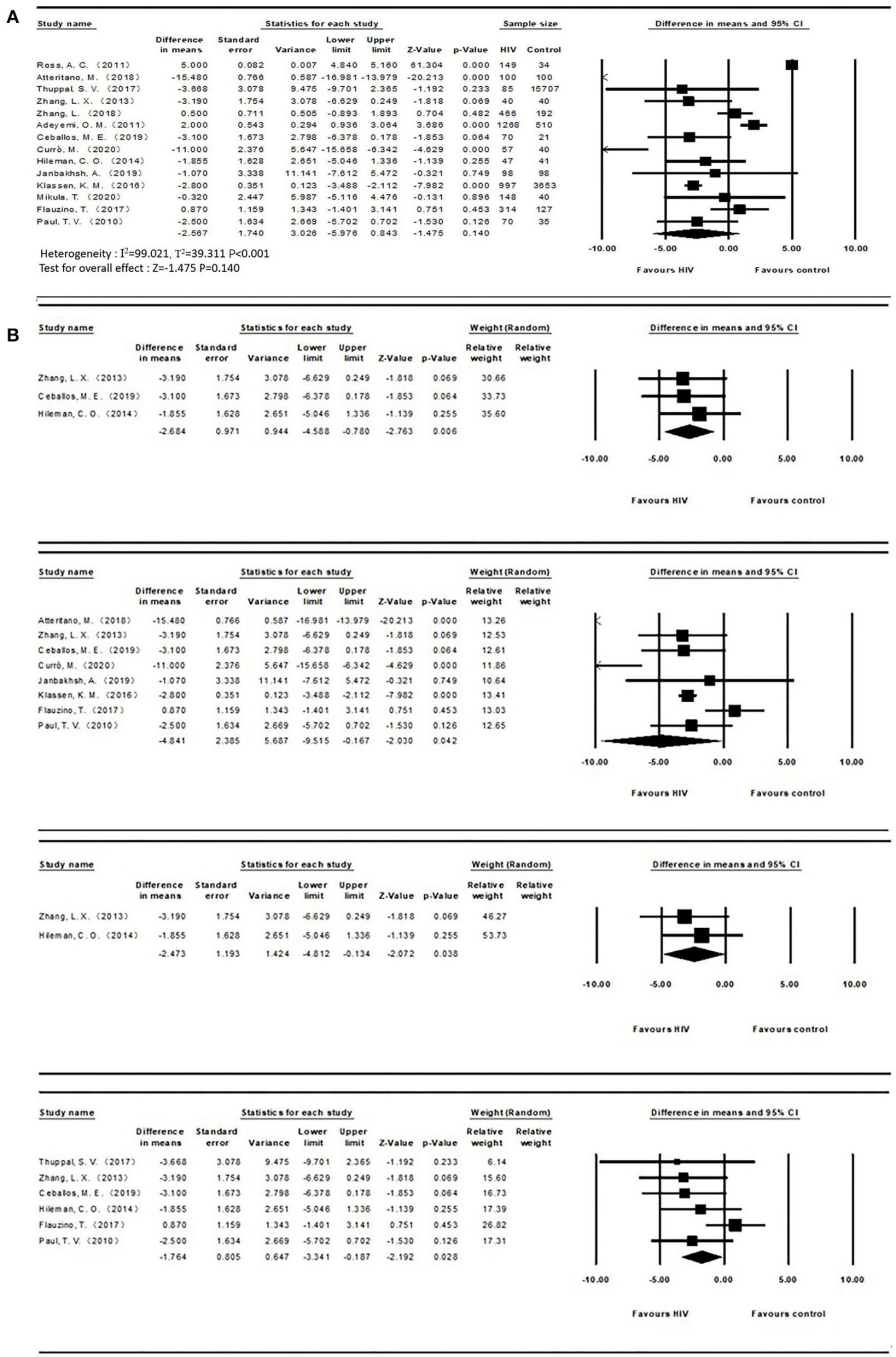
**(A)** Vitamin D levels in HIV and control groups; **(B)** Subgroups of vitamin D levels (ART-naïve, Latitude ≤40, detectable HIV RNA, Age ≤ 40 from top to bottom) between both groups.

**Figure 4:**
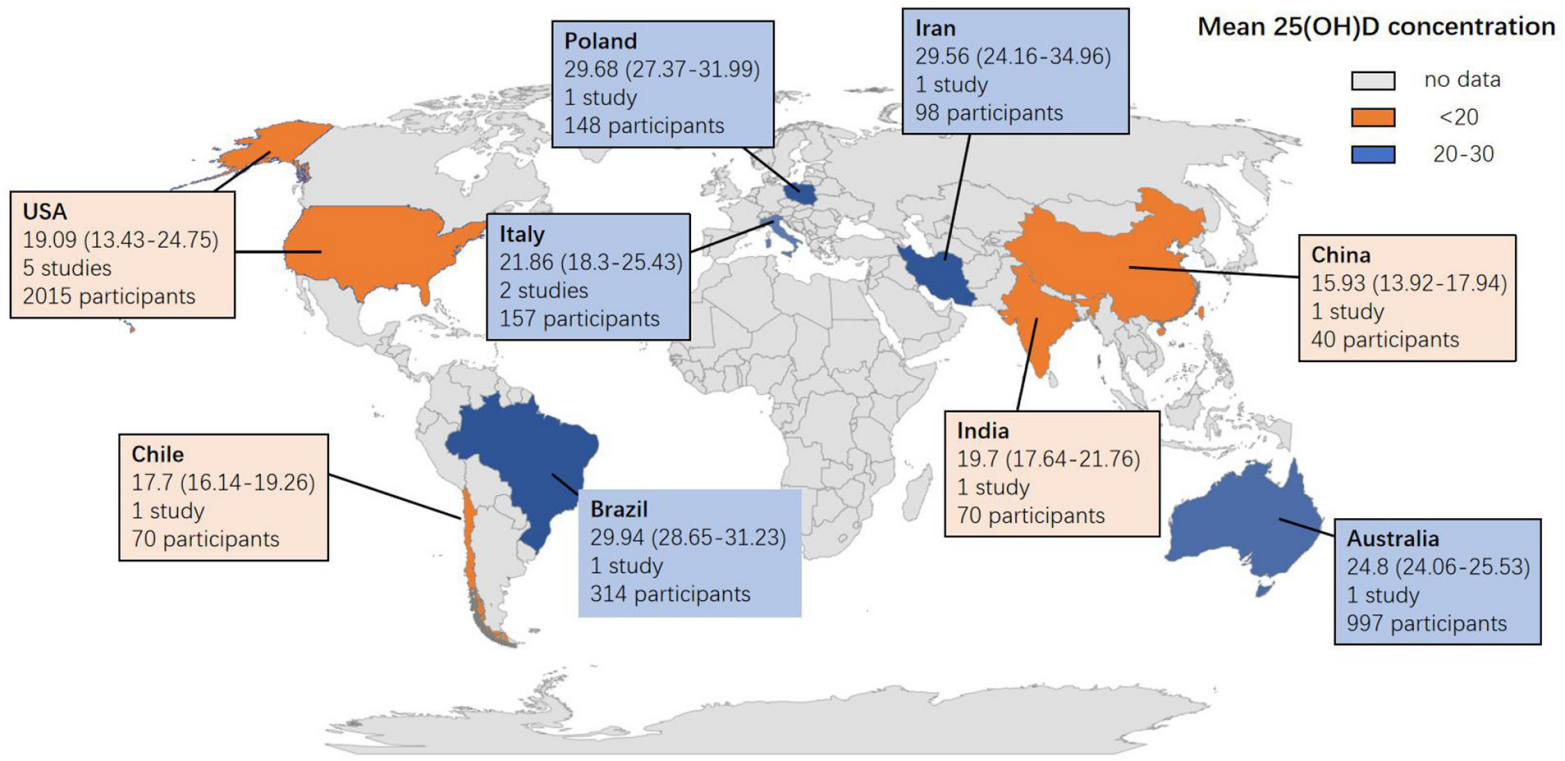
Global map for vitamin D concentration in HIV individuals from 14 included studies. Data are the mean (95% CI) 25 (OH) D concentrations reported in studies done in each country. Pooled means were calculated if the country had more than one study. Twenty five (OH) D = 25-hydroxyvitamin D.

The mean and SD of serum 25(OH)D levels reported by 14 studies were pooled and computed. The overall mean difference between HIV and control groups was −2.567 (95% CI, −5.976 to 0.843; *P* = 0.140), with substantial heterogeneity (*I*^2^ = 99.021). In subgroup analyses, the overall mean difference (MD) was −1.876 (95% CI, −3.341 to −0.187; *P* = 0.028; *I*^2^ = 27.956) from 6 studies (age less than or equal to 40), −2.684 (95% CI, −4.588 to −0.780; *P* = 0.006; *I*^2^ = 0.000) from 3 studies (ART-naïve HIV participants), −4.841 (95% CI, −9.515 to −0.167; *P* = 0.042; *I*^2^ = 97.326) from 8 studies (latitude less than or equal to 40) and −2.473 (95% CI, −4.812 to −0.134; *P* = 0.038; *I*^2^ = 0.000) from 2 studies (HIV participants with detectable HIV RNA). It was asymmetric in the visual funnel plot. Therefore, we performed Duval and Tweedies's trim and fill test and found that the summary MD was still not significantly different between the HIV and control groups after 4 studies were trimmed and filled. The forest plots are shown in Figure 3. Detailed subgroup results and the funnel plots are shown in Supplementary Table 3 and Supplementary Figure 2. The results of meta-regression for both outcomes are presented in Supplementary Table 4. The global map for vitamin D levels in HIV individuals from included studies is shown in Figure 4.

## Discussion

This review is the first to quantitatively and systematically compare differences in VDD prevalence and serum VD levels between PLWH and the general population. The HIV group had a lower but not significantly different vitamin D level than the control group. In contrast, a higher vitamin D deficiency prevalence tended to occur in PLWH.

### Principal Findings and Comparison to Other Studies

Observational studies conducted only on HIV participants reported high vitamin D deficiency prevalence. Some mechanisms have been explored to interpret the relationship between HIV and VDD. PLWH have many more risk factors than traditional patients. For example, ART is related to vitamin D deficiency (42). Avihingsanon et al. reported that EFV use was an independent predictor of VDD (43). Another prospective study observed lower vitamin D levels after 21 months of treatment with PI or NRTIs (44). These results were consistent with the outcomes of our subgroups stratifying HIV participants into ART-experienced and ART-naïve. Interestingly, a study conducted in veterans also demonstrated a higher prevalence in HIV participants than in the control group (53.2 vs. 38.5%, *p* < 0.001), yet tenofovir use was inversely associated with vitamin D deficiency (OR 0.72, 95% CI 0.54–0.96) (32). Likewise, Adeyemi reported an opposite effect of recent PI use (18). The disagreement regarding the effect of ART drugs on vitamin D metabolism is still unresolved (18). Unfortunately, we could not perform subgroup analysis stratifying detailed ART regimens due to limited information.

Vitamin D modulates immune responses. Its receptors are expressed on nearly all immune cells, primarily CD8 and CD4 T lymphocytes (45). In addition to responding to infection, it also prevents the development of autoimmune diseases, such as type 1 diabetes, systemic lupus erythematosus, and inflammatory bowel disease. Several studies have evaluated the association between CD4 T cell count and VD status, often with contradictory results. In a pearl study, vitamin D status precART was linked to CD4 T cell recovery (46). The same effect was observed in another longitudinal study stratifying HIV participants into VDD, Vitamin D insufficiency (VDI), and Vitamin D sufficiency (VDS) groups. CD4 count was screened at every visit (47). The VDD and VDI groups displayed lower absolute T cell gain at all time. Likewise, Adeymi and colleagues reported ORs of 2.77 (1.29–6.68) and 1.66 (1.15–2.51) in CD4 <50 and ranged between 50 and 200 in regard to vitamin D deficiency (18). Mikula and colleagues found that vitamin D levels were positively correlated with CD4 percentage (*r* = 0.17, *P* = 0.036) (39). In contrast, Flauzino et al. found no significant difference in CD4 count (categorized into <200, 200–500, and over 500 groups) when stratified by vitamin D levels (vitamin D < 30 ng/ml vs. ≥ 30 ng/ml) (*P* = 0.426) (40). Janbakhsh et al. also demonstrated that CD4 count was not associated with vitamin D levels (28). Similarly, our review did not find a significant association on either outcome by subgrouping according to CD4 count.

Its deficiency is linked with IRIS, HIV disease progression, virologic failure, and death. A longitudinal study supplied healthy participants with high VD3 dosage (50,000 IU/week) for 6 weeks (48). The obtained PBMCs were cultured and infected with HIV at each of the visits. P24 antigen levels were high at initiation and decreased after 6 weeks of VD3 supplementation. The effect of limiting HIV replication was observed through this study, yet it was conducted using healthy PBMCs *in vitro*. In our subgroup of 2 studies in which HIV participants had detectable HIV RNA, HIV participants presented with lower vitamin D levels. Beyond our hypothesis, Flauzino et al. observed a positive correlation between vitamin D level and viral load (when vitamin D level was over or equal to 30 ng/ml) (*r* = 0.178, *p* = 0.039), which may result from increased immune stimulation (40).

The influence of traditional factors involving latitude, BMI, and age is apparent in PLWH. The initial step of VD metabolism occurs in the skin, in which sunlight plays an important role. Adequate sunlight is easy to obtain in lower latitude places. At the same time, a meta-analysis reported a higher prevalence of VDD in Africa. Several factors, such as lifestyle, skin color, and nutritional status could be sources of VDD in this review. In our analysis, the HIV group has a higher risk of VDD at lower latitudes. As reported by Maggi et al., HIV participants were prone to use sunblock to protect their skin because they were young with more frequent outdoor activities and more easily developed malignancies (49). Canuto et al. reported the relationship between sunscreen use and lower vitamin D concentration in PLWH in low latitude areas (50). One study from our review reported a higher use of sunblock among PLWH (*P* = 0.006) (37). The influence of skin color could not be excluded due to lack of detailed information. In addition, PLWH are likely to experience appetite loss, nutrient malabsorption, and inadequate dietary intake (51). A cross-sectional study demonstrated that over 85% of subjects in PLWH had at least one gastrointestinal abnormality (52). Poles et al. reported a high incidence of fat malabsorption in HIV participants (53). Moreover, aging-related VDD is a combined result of all the factors mentioned above. Sunlight exposure, nutrient intake and absorption, and VD synthesis are reduced in elderly patients (54). In addition, Inflammation increases with aging in the imbalanced immune system and the accumulation of senescent cells. PLWH may experience persistent inflammation and immune activation, even with successful viral suppression. Gut-associated lymphoid tissue is damaged early during HIV infection, followed by the translocation of microbial products into the bloodstream, leading to chronic immune activation (55, 56). In addition, HIV shares several proinflammatory biomarkers with aging (55). Vitamin D has antioxidant and anti-inflammatory effects. However, the relationship that occurs first between vitamin D and aging is still unclear (57).

PLWH are prone to present decreased bone mass. Vitamin D also functions in bone metabolism with calcium. Shahar and colleagues reported that vitamin D status was associated with bone mineral density in female PLWH (58). In addition, Atteritano et al. demonstrated a statistical association between vitamin D insufficiency and vertebral fracture (OR = 9.15, *p* < 0.04) (34).

Therefore, PLWH need to be screened to identify the potential vitamin D deficiency. Additional randomized controlled studies must perform due to the unclear suitable dosage of vitamin D supplements in HIV participants. Moreover, we advise establishing a model predicting and screening PLWH at higher risk for VD deficiency, which could act in a positive way in detecting potential deficiency in resource-limited places. Appropriate cutoffs of vitamin D deficiency should also be considered due to the differential current criteria developed based on bone-related studies.

### Limitations

Several points should be considered in interpreting our results. First, we only adopted the baseline data into our meta-analysis from all the literature. We could not obtain a causal association from these studies because the change in vitamin D may interfere with lifestyle. Second, it should be noted that several studies did not survey detailed information on vitamin D supplements. Third, in our review, the included studies were conducted in Europe, the middle and east of Asia, America, and Australia. Therefore, it is unclear whether this association exists elsewhere. Our included studies were also limited to the English language. Finally, age, sex, type of HIV participants, latitude, CD4 counts, publication year and BMI could be sources of heterogeneity, which was supported by subgroup analysis and meta-regression.

## Conclusions

In conclusion, in our systematic review and meta-analysis, we observed a modest but significantly higher vitamin D deficiency prevalence in PLWH than in control subjects. Furthermore, HIV participants were prone to VDD in when receiving ART, living in lower latitudes, being older, having a lower BMI, and being male. In addition, we strongly recommend performing further cohort studies and population-based trials to confirm our results.

## Data Availability Statement

The original contributions presented in the study are included in the article/Supplementary Material, further inquiries can be directed to the corresponding author/s.

## Author Contributions

YWa: article review, quality assessment, and draft written. XH: article review, quality assessment, and manuscript revise. YWu and AL: data extraction and quality assessment. YT, TZ, and HW: manuscript revise. MR: article review. WW: study design, article review, quality assessment, and manuscript revise. All authors contributed to the article and approved the submitted version.

## Conflict of Interest

The authors declare that the research was conducted in the absence of any commercial or financial relationships that could be construed as a potential conflict of interest.

## Publisher's Note

All claims expressed in this article are solely those of the authors and do not necessarily represent those of their affiliated organizations, or those of the publisher, the editors and the reviewers. Any product that may be evaluated in this article, or claim that may be made by its manufacturer, is not guaranteed or endorsed by the publisher.

## Footnotes

^1^Available online at: https://www.unaids.org/en (accessed January 15, 2021) (2019).

## Abbreviations

ART: Antiretroviral therapy
HIV: Human immunodeficiency virus
PLWH: People Living with HIV
VDD: Vitamin D deficiency
SE: Standard error
SD: Standard difference
IQR: Interquartile range
MD: Mean difference
OR: Odds ratio
BMI: Body Mass Index
ELISA: Enzyme-linked immunosorbent assay
TDF: Tenofovir Disoproxil Fumarate
TAF: Tenofovir alafenamide
VDR: Vitamin D receptor
EFV: Efavirenz
PI: Protease inhibitor
VDI: Vitamin D insufficiency
VDS: Vitamin D sufficiency
UNAIDS: Joint United Nations Programme on HIV/AIDS
NRTI: the nucleoside reverse transcriptase inhibitor.
