# Supplementary material for: Increased Risk of Vitamin D Deficiency Among HIV-Infected Individuals: A Systematic Review and Meta-Analysis
Source: Front Nutr. 2021 Aug 18;8:722032. doi: 10.3389/fnut.2021.722032 (PMC8418196; doi:10.3389/fnut.2021.722032)
Supplement: Supplementary file 1 [file Data_Sheet_1.pdf]

Table S1 Search Strategy used in four databases

**Literature search strategy in PubMed: 789**
1: "HIV"[MeSH Terms] OR "HIV Infections"[MeSH Terms] OR "hiv infection"[Title/Abstract] OR "human immunodeficiency virus"[Title/Abstract] OR "aids virus"[Title/Abstract] OR "acquired immune deficiency syndrome virus"[Title/Abstract] OR "acquired immunodeficiency syndrome"[Title/Abstract] OR "HIV"[Title/Abstract]
2: "Vitamin D Deficiency"[MeSH Terms] OR "Vitamin D"[MeSH Terms] OR "Vitamin D"[Title/Abstract] OR "25 hydroxyvitamin d"[Title/Abstract] OR "1,25 dihydroxyvitamin d"[Title/Abstract] OR "Vitamin D Deficiency"[Title/Abstract] OR "Ergocalciferols"[Title/Abstract] OR "Calcifediol"[Title/Abstract] OR "Cholecalcifero"[Title/Abstract]
3: 1 AND 2
**Literature search strategy in Web of Science: 721**
1: Search TS = (HIV) OR (Human Immunodeficiency Virus) OR (AIDS Virus) OR (Acquired Immune Deficiency Syndrome Virus) OR (Acquired Immunodeficiency Syndrome) OR (hiv infection)
2: Search TS = (Vitamin D) OR (Vitamin D Deficiency) OR (25 hydroxyvitamin d) OR (1,25 dihydroxyvitamin d) OR (Ergocalciferols) OR (Calcifediol) OR (Cholecalciferol)
3: 1 AND 2
5: Exclude (LETTER OR EDITORIAL MATERIAL OR NOTE OR REVIEW OR BOOK CHAPTER OR CORRECTION OR BOOK OR MEETING ABSTRACT OR PROCEEDINGS PAPER OR EARLY ACCESS)
**Literature search strategy in EMBASE: 3134 (1717)**
1: 'hiv infection':ti,ab,kw OR 'human immunodeficiency virus':ti,ab,kw OR 'aids virus':ti,ab,kw OR 'acquired immune deficiency syndrome virus':ti,ab,kw OR 'acquired immunodeficiency syndrome':ti,ab,kw OR hiv:ti,ab,kw
2: 'human immunodeficiency virus'/exp；
3: 'human immunodeficiency virus infection'/exp
4: 1 OR 2 OR 3
5:'vitamin d':ti,ab,kw OR '25 hydroxyvitamin d':ti,ab,kw OR '1,25 dihydroxyvitamin d':ti,ab,kw OR 'vitamin d deficiency':ti,ab,kw OR ergocalciferols:ti,ab,kw OR calcifediol:ti,ab,kw OR cholecalcifero:ti,ab,kw
6: 'vitamin d'/exp
7: 'vitamin d deficiency'/exp
8: 5 OR 6 OR 7 OR
9: 4 AND 8

Exclude ([conference abstract]/lim OR [conference paper]/lim OR [conference review]/lim OR [letter]/lim OR [note]/lim OR [review]/lim)
**Literature search strategy in Cochrane library: 294**
1: hiv:ti,ab,kw OR 'human immunodeficiency virus':ti,ab,kw OR 'aids virus':ti,ab,kw OR 'acquired immune deficiency syndrome virus':ti,ab,kw OR 'acquired immunodeficiency syndrome':ti,ab,kw OR 'hiv infection':ti,ab,kw
2: **MeSH descriptor [HIV**] OR **MeSH descriptor [HIV infection**]
3: 1 OR 2
4: 'vitamin d':ti,ab,kw OR '25 hydroxyvitamin d':ti,ab,kw OR '1,25 dihydroxyvitamin d':ti,ab,kw OR 'Vitamin D Deficiency':ti,ab,kw OR 'Ergocalciferols':ti,ab,kw OR 'Calcifediol':ti,ab,kw OR 'Cholecalcifero':ti,ab,kw
5: **MeSH descriptor [**Vitamin D] OR **MeSH descriptor [**Vitamin D deficiency]
6: 4 OR 5
7: 3 AND 6

Table S2 subgroup analysis of VDD prevalence deference between HIV and control groups

Subgroup category	Number	Point estimate(95%CI)	P	I^2	T^2	P
Age>40	7	1.647(1.020-2.659)	0.041	94.568	0.332	<0.001
Age≤40	4	1.217(0.712-2.080)	0.474	49.552	0.146	0.114
BMI>25	5	1.057(0.556-2.011)	0.865	94.757	0.457	<0.001
BMI≤25	2	2.120(1.122-4.008)	0.021	0.000	0.000	0.988
latitude>40	3	1.331(0.695-2.549)	0.388	37.828	0.125	0.200
latitude≤40	7	1.805(1.373-2.372)	<0.001	74.576	0.070	0.001
CD4count>500	5	1.493(0.858-2.596)	0.156	82.646	0.290	<0.001
CD4count≤500	5	1.322(0.681-2.566)	0.409	94.401	0.475	<0.001
Sex(female)	1	0.585(0.468-0.731)	<0.001	0.000	0.000	1.000
Sex(male)	2	2.120(1.122-4.008)	0.021	0.000	0.000	0.998
ART-experienced	3	2.296(1.287-0.497)	0.005	19.927	0.053	0.287
ART-naive	2	1.177(0.379-3.657)	0.778	62.765	0.420	0.101
Developed country	7	1.520(0.921-2.510)	0.101	94.723	0.353	<0.001
Developing country	4	1.377(0.897-2.114)	0.144	42.282	0.079	0.158

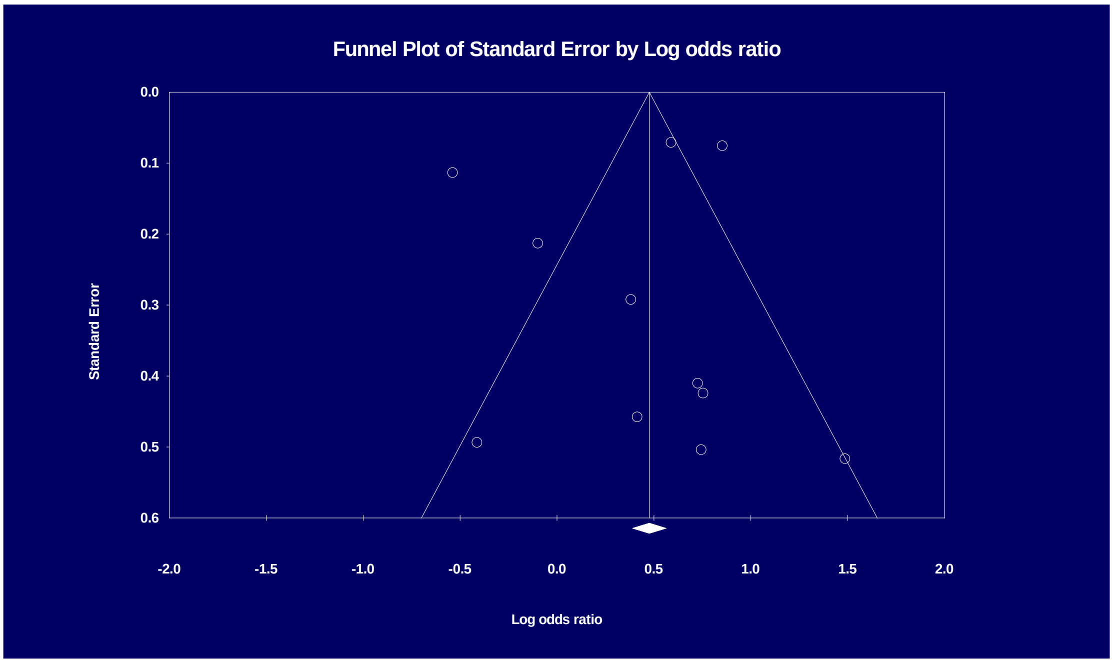

Figure S1 funnel plot of VDD prevalence difference between HIV and control groups

Study name	Statistics with study removed				
	Point	Lower limit	Upper limit	Z-Value	p-Value
Ross.A.C (2011)	1.462	0.976	2.190	1.840	0.066
Adeyemi （2011）	1.713	1.333	2.200	4.209	0.000
Celallos （2019）	1.466	0.982	2.189	1.871	0.061
Curro （2020）	1.391	0.937	2.065	1.636	0.102
Hidron （2015）	1.492	0.890	2.500	1.519	0.129
Hileman （2014）	1.596	1.073	2.373	2.308	0.021
Janbakhsh （2019）	1.508	0.999	2.277	1.956	0.051
Klassen （2016）	1.424	0.917	2.212	1.572	0.116
Mikula （2020）	1.502	1.004	2.247	1.980	0.048
Flauzino （2017）	1.597	1.060	2.405	2.241	0.025
Paul （2010）	1.459	0.975	2.185	1.835	0.067
	1.502	1.023	2.205	2.078	0.038

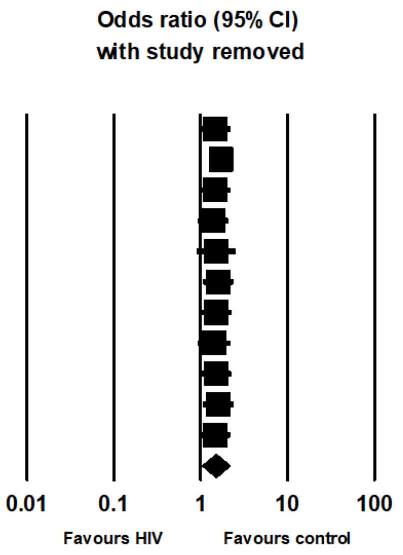

Vtamin D deficiency prevalence

Figure S2 sensitivity analysis of included 11 studies

Table S3 subgroup analysis of VD levels deference between HIV and control groups

Subgroup category	Number	Point estimate(95%CI)	P	I^2	T^2	P
Age>40	8	-2.849(-7.496-1.797)	0.229	99.437	42.231	<0.001
Age≤40	6	-1.764(-3.341--0.187)	0.028	27.956	1.071	0.225
BMI>25	5	1.201(-1.407-3.810)	0.367	94.040	6.989	<0.001
BMI≤25	4	-6.149(-14.103-1.804)	0.130	97.091	63.598	<0.001
latitude>40	3	1.188(-4.044-6.420)	0.656	91.061	18.770	<0.001
latitude≤40	8	-4.841(-9.515--0.167)	0.042	97.326	42.293	<0.001
Undetectable HIV RNA	1	-0.320(-5.116-4.476)	0.896	0.000	0.000	1.000
Detectable HIV RNA	2	-2.473(-4.812--0.134)	0.038	0.000	0.000	0.577
Sex(female)	1	2.000(0.936-3.064)	<0.001	0.000	0.000	1.000
Sex(male)	3	-1.340(-3.884-1.205)	0.302	65.881	3.307	0.053
ART-experienced	4	-0.918(-5.457-3.621)	0.692	96.633	18.722	<0.001
ART-naive	3	-2.684(-4.588--0.780)	0.006	0.000	0.000	0.817
Developed country	9	-2.983(-7.336-1.369)	0.179	99.366	41.797	<0.001
Developing country	5	-1.618(-3.514-0.279)	0.095	37.175	1.695	0.173

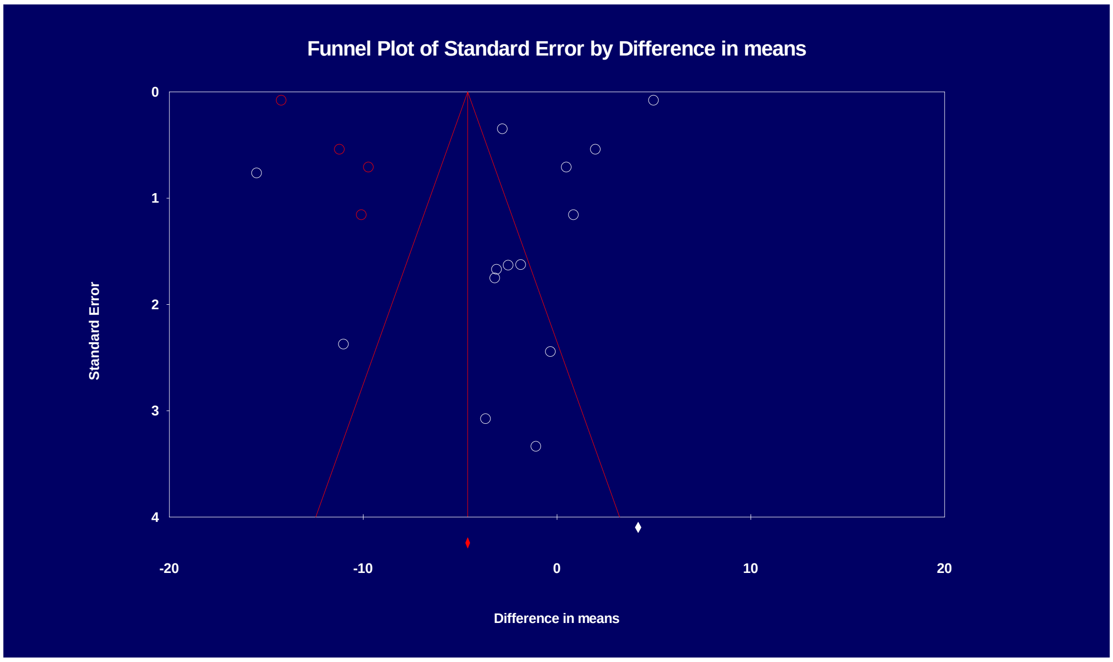

Figure S3 funnel plot of VD levels difference between HIV and control groups

Study name	Point	Standard error	Variance	Lower limit	Upper limit	Z-Value	p-Value
	Statistics with study removed						
Ross, A. C.（2011）	-3.195	1.582	2.501	-6.295	-0.096	-2.020	0.043
Atteritano, M.（2018）	-1.418	1.372	1.882	-4.107	1.271	-1.034	0.301
Thuppal, S. V.（2017）	-2.494	1.797	3.231	-6.017	1.029	-1.387	0.165
Zhang, L. X.（2013）	-2.519	1.809	3.274	-6.065	1.027	-1.392	0.164
Zhang, L.（2018）	-2.822	1.889	3.570	-6.525	0.881	-1.494	0.135
Adeyemi, O. M.（2011）	-2.949	1.978	3.911	-6.825	0.927	-1.491	0.136
Ceballos, M. E.（2019）	-2.526	1.810	3.278	-6.074	1.023	-1.395	0.163
Currò, M.（2020）	-1.958	1.783	3.181	-5.453	1.538	-1.098	0.272
Hileman, C. O.（2014）	-2.622	1.815	3.294	-6.180	0.935	-1.445	0.148
Janbakhsh, A.（2019）	-2.662	1.797	3.231	-6.185	0.860	-1.481	0.139
Klassen, K. M.（2016）	-2.556	1.979	3.917	-6.436	1.323	-1.292	0.196
Mikula, T.（2020）	-2.728	1.807	3.265	-6.269	0.814	-1.510	0.131
Flauzino, T.（2017）	-2.844	1.838	3.379	-6.447	0.758	-1.547	0.122
Paul, T. V.（2010）	-2.572	1.813	3.287	-6.125	0.981	-1.419	0.156
	-2.567	1.740	3.026	-5.976	0.843	-1.475	0.140

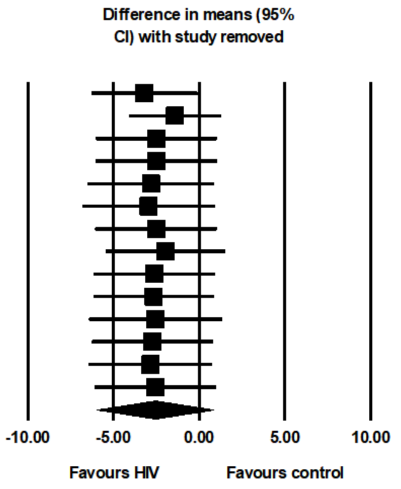

Figure S4 sensitivity analysis of included 14 studies

Table S4 Meta-regression for heterogeneity

Moderators	Number	I^2	P	Variance explained (%)
Studies for evaluating VDD prevalence				
Continuous				
Male proportion	11	91.8	<0.001	30
BMI	7	92.37	<0.001	52
Undetectable HIV RNA proportion	7	94.89	<0.001	7
Studies for evaluating VD level				
Categorical				
ART	14	99.02	<0.001	20
latitude	14	99.22	<0.001	28
BMI	9	99.03	<0.001	73
CD4 count	14	99.02	<0.001	40
Continuous				
Undetectable HIV RNA proportion	7	98.89	<0.001	31
Publication year	14	99.02	<0.001	52
